# Epicardial adipose tissue is associated with higher recurrence risk after catheter ablation in atrial fibrillation patients: a systematic review and meta-analysis

**DOI:** 10.1186/s12872-022-02703-9

**Published:** 2022-06-11

**Authors:** Jun Chen, Ziwei Mei, Yang Yang, Chuxing Dai, Yimin Wang, Rui Zeng, Qiang Liu

**Affiliations:** 1 The Third Affiliated Hospital of Zhejiang Chinese Medical University, Hangzhou, 310000 Zhejiang China; 2 Zhejiang Chinese Medical University, Hangzhou, 310000 Zhejiang China; 3 Lishui Hospital, The Fifth Affiliated Hospital of Wenzhou Medical University, Lishui, 323000 Zhejiang China

**Keywords:** Atrial fibrillation, Epicardial adipose tissue, Recurrence risk, Catheter ablation, Meta-analysis

## Abstract

**Objective:**

Our study aimed to investigate the association between epicardial adipose tissue (EAT) and atrial fibrillation (AF) recurrence risk after catheter ablation.

**Methods:**

We searched PubMed, Embase, and Cochrane Library databases up to November 30, 2021 without language restrictions. Outcome was the relative risk (RR) of EAT contributes to AF recurrence after catheter ablation. The RR and 95% confidence interval (CI) was pooled by the random-effect model.

**Results:**

10 studies that contained 1840 AF patients were included in our study. The result of this study showed that EAT amount was associated with higher risk of AF recurrence after catheter ablation (RR = 1.06, 95% CI 1.02–1.11, P = 0.005) and EAT related thickness was a risk factor for AF recurrence after catheter ablation (RR = 1.73, 95% CI 1.04–2.87, P = 0.040). Sub-analysis showed that EAT was strongly associated with higher risk of AF recurrence common in Asian population (RR = 1.25, 95% CI 1.10–1.43, P < 0.001), patients aged ≤ 60 years old (RR = 2.01, 95% CI 1.18–3.44, P = 0.010), and follow-up more than 1 year (RR = 1.06, 95% CI 1.01–1.11, P = 0.020).

**Conclusion:**

The meta-analysis demonstrated that EAT related thickness seems to be the marker most strongly associated with a greater risk of AF recurrences after catheter ablation. It should be included into risk stratification for predicting AF recurrent before catheter ablation.

**Supplementary Information:**

The online version contains supplementary material available at 10.1186/s12872-022-02703-9.

## Introduction

Atrial fibrillation (AF) is the most common cardiac arrhythmia worldwide, it has been estimated that approximately 6–12 million people will develop AF in the US by 2050 and 17.9 million people will develop AF in Europe by 2060 [1], which contributes to the increased cardiovascular and cerebrovascular disorder, lead to huge health care costs and public health burden worldwide [2]. It is widely recognized that AF ablation is effective in controlling AF and its associated symptoms [3]. Recently, Poole JE et al. reported that compared with drug therapy, catheter ablation was effective in reducing recurrence of any AF by 48% and symptomatic AF by 51% over a 5-years follow-up [4]. However, from another point of view, there was still a high recurrence risk after catheter ablation (49.9%) over 5 years of follow-up [4]. It was reported that the success rate of catheter ablation was only 70% for paroxysmal AF and 50% for persistent AF [5]. Early identification of such AF patients with high recurrence risk after ablation has implications for improving the prognosis of AF patients.

Epicardial adipose tissue (EAT), also referred to epicardial fat tissue (EFT), is located between the myocardium and the visceral pericardium and is commonly found in the atrioventricular and inter-ventricular grooves of the adult human heart (i.e. pericardial fat, perivascular fat, and myocardial steatosis) [6]. Previous studies reported that the increase of EAT amount was associated with higher occurrence of AF [7–11]. Gaeta M et al. conducted a meta-analysis comparing EAT volume in healthy subjects and AF patients, which confirmed that a statistical difference of EAT volume among persistent AF, paroxysmal AF, and healthy subjects [12].

Several previous retrospective studies reported that EAT was also associated with higher recurrence risk in AF patients after catheter ablation [13–16]. However, recently, El Mahdiui M et al. study showed that there was no significant association between the posterior left atrial adipose tissue amount and AF recurrence risk after catheter ablation (HR = 1.01, 95% CI 0.97–1.01, P = 0.759) [17]. This find was similar to the result of Romanov et al. study that no statistically significant association between either total or peri-atrial EAT volumes and AF recurrence risk after a 12 months follow-up (HR = 1.02, 95% CI 0.99–1.05, P = 0.11) [18]. These results were still controversial to date. To clarify this question, we conducted a systematic review and meta-analysis to investigate the association between EAT and AF recurrence after catheter ablation.

## Material & methods

This systematic review was conducted following the recommendations of the Cochrane Collaboration Handbook, observational studies in epidemiology statement [19], Meta-Analysis and Systemic Reviews of Observational Studies (MOOSE) [20], and Preferred Reporting Items for Systematic Review and Meta-Analysis (PRISMA) [20]. The Preferred Reporting Items for Systematic Review and Meta-Analysis Protocols (PRISMA-P) were showed in Additional file 1.

### Search strategy

**Fig. 1:**
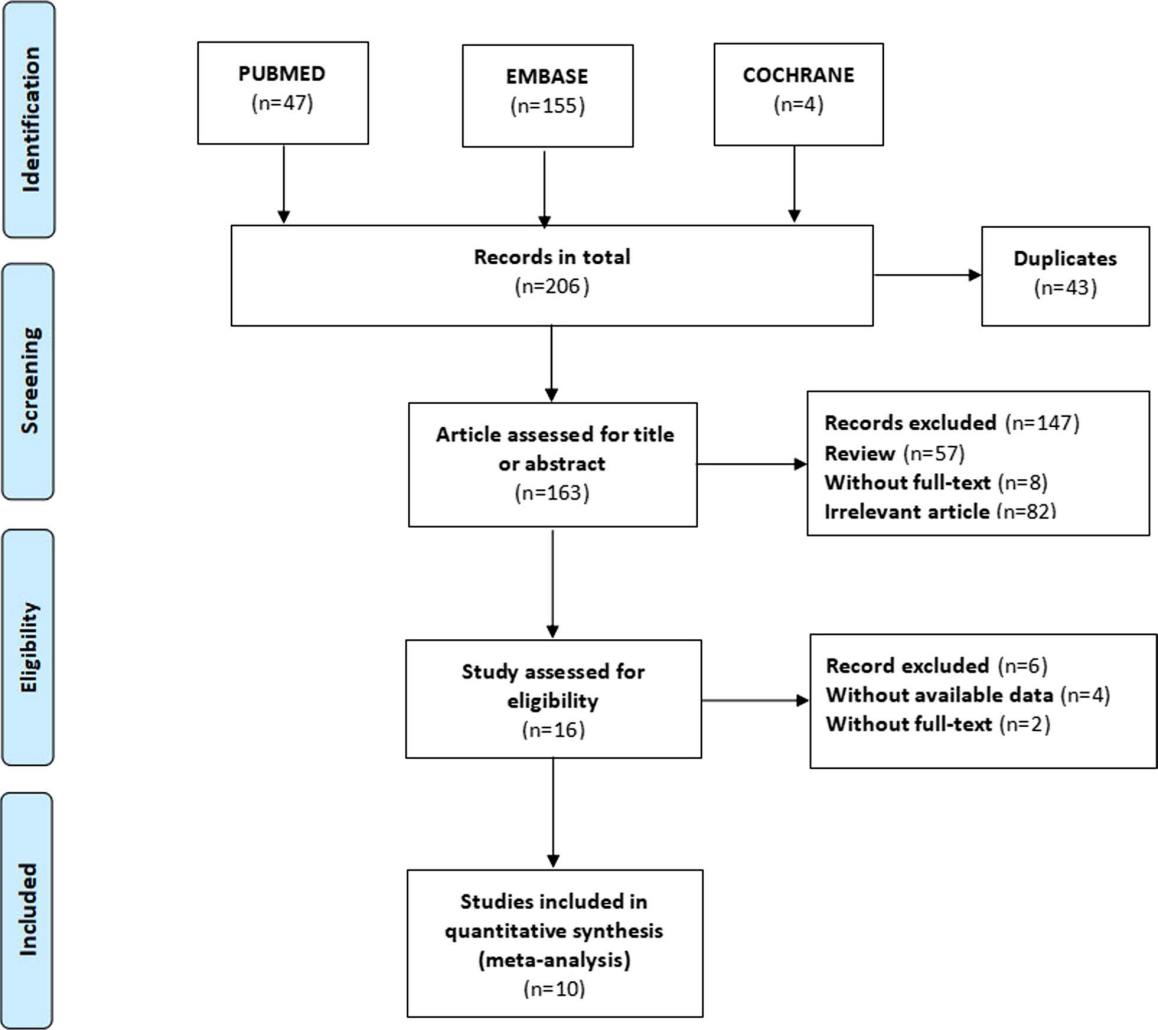
The process of study selection

We searched the databases PubMed, Embase, and Cochrane Library until November 30, 2021. Search keywords were “atrial fibrillation,” “AF,” “epicardial adipose tissue,” “epicardial fat,” “catheter ablation,” and “radiofrequency ablation,”. We also searched two clinical trials registers as well as previous systematic reviews and reference lists of included studies. No language restrictions were applied. Our search strategy combined text words and subject headings, with details presented in Additional file 2. This study was completed in accordance with the guidelines of Preferred Reporting Items for Systematic Reviews and Meta-Analyses (PRISMA) [20], the study selection process is shown in Fig. 1.

### Inclusion and exclusion criteria

We included studies of all designs that produce estimates of test accuracy or provide data from which we can compute estimates, including the following: (1) Reported relevant EAT parameter (e.g., EAT volume, EAT area, EAT thickness) or with other statistical indexes (e.g., EAT volume index, Left-atrial EAT index, peri-atrial EAT thickness/Total EAT thickness); (2) Available data of relative risk (RR), or hazard ratio (HR) and their 95% confidence intervals (CI) for EAT contribute to AF recurrence risk after catheter ablation; (3) Follow-up more than 6 months; Exclusion criteria including the following: (1) For duplicated reports, the last published study was included; (2) Conference papers without available data; (3) Not catheter ablation for AF.

### Study selection and data extraction

Three independent reviewers (MZW, YY, and WYM) screened titles, abstracts for potential eligible studies, and assessed full-text articles against the eligibility criteria. Disagreements were resolved through consensus with the fourth reviewer (LQ). Data was extracted including author, study type, region, study size, the measurement of EAT, the interesting indicator (eg., adjusted relative risk, or adjusted hazard ratio) of each study included as the main outcomes. The baseline characteristic of each study population was also documented.

### Study quality assessment

The quality of observational studies were evaluate by the Newcastle Ottawa quality assessment scale (NOS) [21]. The assessment elements included the study groups selection, comparability of groups, and ascertainment of the exposure and outcomes. Seven scores or more was regarded as high-quality study.

### Data synthesis and analysis

We used relative risk (RR) and their associated 95% confidence intervals (Cl) to evaluate the association between EAT amount and AF recurrence after catheter ablation, and considered a P value ≤ 0.05 to be statistically significant. The effect size conduction was merged by fixed-effect models or random-effect models according to the heterogeneity (fixed-effect models for low heterogeneity: P > 0.05 or I^2^ < 50%; and random-effect models for high heterogeneity: P ≤ 0.05 or I^2^ ≥ 50%). We performed subgroup analysis to further explore the effect of different factors on the final results. First subgroup analysis investigate which EAT parameters (EAT related volume, EAT related thickness, and EAT related index) were significantly associated with AF recurrence after catheter ablation, other subgroup analysis mainly investigate the source of heterogeneity according to population average age (≥ 60 years old vs. < 60 years old), ethnicity (Asian vs non-Asian), follow-up duration of study (> 1 year vs. ≤ 1 year) and the study sample size (> 200 patients vs. ≤ 200 patients). Sensitivity analysis was conducted to assess the impact of each study on the final results by eliminating one study and meta-merged the left ones. Sequentially deleting the later study and then meta-merged the left ones to investigate every study’s effect on the final results. Publication bias was resented by a funnel plot. Stata 15.0 (StataCorp, College Station, TX, USA) and Review Manager version 5.4 (The Nordic Cochrane Centre, The Cochrane Collaboration, Copenhagen) were used to conduct our data and pool the meta-analysis.

## Results

### Inclusion of studies and quality assessment

Our study included 206 articles in the searching process. Ten articles were included in the current meta-analysis after removing unrelated articles. The specific screening process is demonstrated in Fig. 1. All of these included studies were retrospective cohort studies. Of the ten retrospective cohort studies, nine were regarded as high-quality due to their low risk of bias and representativeness of the exposed cohort. The general information and quality assessment results of included studies were summarized in Table 1. For additional details, please refer to Additional file 3. Among the 1840 AF patients, 1322 (71.8%) patients presented with paroxysmal atrial fibrillation; 518 (28.2%) patients presented with persistent AF. Totally, 630 patients (34.2%) have experienced AF recurrent after catheter ablation. The AF recurrent rate range from 20 to 67.9% among these studies. Almost all these studies measured the EAT with computed tomography (CT), only one study based on echocardiography.

**Table1:** Characteristics of include studies

Author /year	region	Size	Population	Follow-up	Age	Study type	object	AFR	RR/HR	Adjusted	Measure	Quality assessment
Nagashima/2011 [22]	Japan	40	24 PAF 16 Per AF	0.85 y	58.0 y	Retrospective	LA-EAT volumes	15(37.5%)	7.15 (3.03–11.3)	Age, BMI, HbA1c, HDL-C, LDL-C, TG	CT	high-quality
Masuda/2015 [23]	Japan	53	22 PAF 31 Per AF	1.33 y	61.5 y	Retrospective	LA-EAT volumes	36(67.9%)	1.06 (1.03–1.10)	PAF rate, Left atrial volume, Radio-frequency application time	CT	high-quality
Stojanovska/2015 [24]	United States	169	94 PAF 75 Per AF	3.2 y	64.0 y	Retrospective	EAT volumes	78(46%)	1.009 (1.001–1.01)	age, gender, BMI	CT	high-quality
Canpolat/2016 [13]	Turkey	234	190 PAF 44 Per AF	1.7 y	54.0 y	Retrospective	EAT thickness	45(19.2%)	1.36 (1.10–1.66)	Age, Dyslipidemia, Non-paroxysmal AF rate, Duration of AF, EHRA score, LA diameter, hs-CRP	Echo	high-quality
Chao/2013 [14]	China	283	227 PAF 56 Per AF	1.3 y	54.6 y	Retrospective	EAT thickness	95(33.6%)	2.863 (2.112–3.882)	Non-paroxysmal AF rate, CHADS2 score, LA diameter	Echo/CT	high-quality
Maeda/2018 [25]	Japan	218	143 PAF 78 Per AF	1.45 y	64.0 y	Retrospective	EATVi	61(28%)	1.02 (1.00–1.03)	Non-paroxysmal AF rate, Body height, eGFR, IVS thickness, Septal E/E’	CT	high-quality
Sanghai/2018 [15]	United States	274	189 PAF 85 Per AF	1.0 y	61.0 y	Retrospective	iLAEAT	109(40%)	2.93 (1.34–6.43)	CHA2DS2Vascscore BMI, LA Volume, LV mass index, average E/e’	CT	high-quality
Kawasaki/2020 [16]	Japan	64	64 PAF	1.0 y	70.7 y	Retrospective	peri-atrial EAT/TEA thickness	14(21.9%)	4.822(1.209–32.809)	Calcium channel blockers, Delta WR	CT	high-quality
Mahdiui/2021 [17]	Hungary	460	354 PAF 106 Per AF	1.5 y	61.0 y	Retrospective	Posterior LAEA thickness	168(37%)	1.01 (0.97–1.04)	Age, sex, AF type, BMI, antiarrhythmic drugs, LVEF < 50%, E/A-ratio, LA volume	CT	high-quality
Romanov/2021 [18]	Russian	45	15 PAF 30 Per AF	1.0 y	55.2 y	Retrospective	peri-atrial EAT volumes	9(20%)	1.02 (0.99–1.05)	NA	CT	medium-quality

*PAF* paroxysmal atrial fibrillation, *Per AF* persistent AF, *Echo* Echocardiographic *CT* computed tomography, *EATV index* epicardial adipose tissue volume index, *LA-EAT* left atrial epicardial adipose tissue, *TEAT* total epicardial adipose tissue, *EATT* epicardial adipose tissue thickness, *BMI*, body mass index, *HDL-C* high density lipoprotein cholesterol, *LDL-C* low density lipoprotein cholesterol, *TG* triglycerides, *EHRA* european heart rhythm association, *LVEF* left ventricular ejection fraction, *WR* washout rate, *E/e’* mitral inflow velocity (E)/mitral annular velocities (e’), *eGFR* estimated glomerular filtration rate, *IVS* interventricular septal, *EATVi* EATV index (EATVI: EATV/body surface area, mL/m^2^), *iLAEAT* index LAEAT (iLAEAT: LAEAT/body surface area, mL/m^2^)

### Results of the meta-analysis

**Fig. 2:**
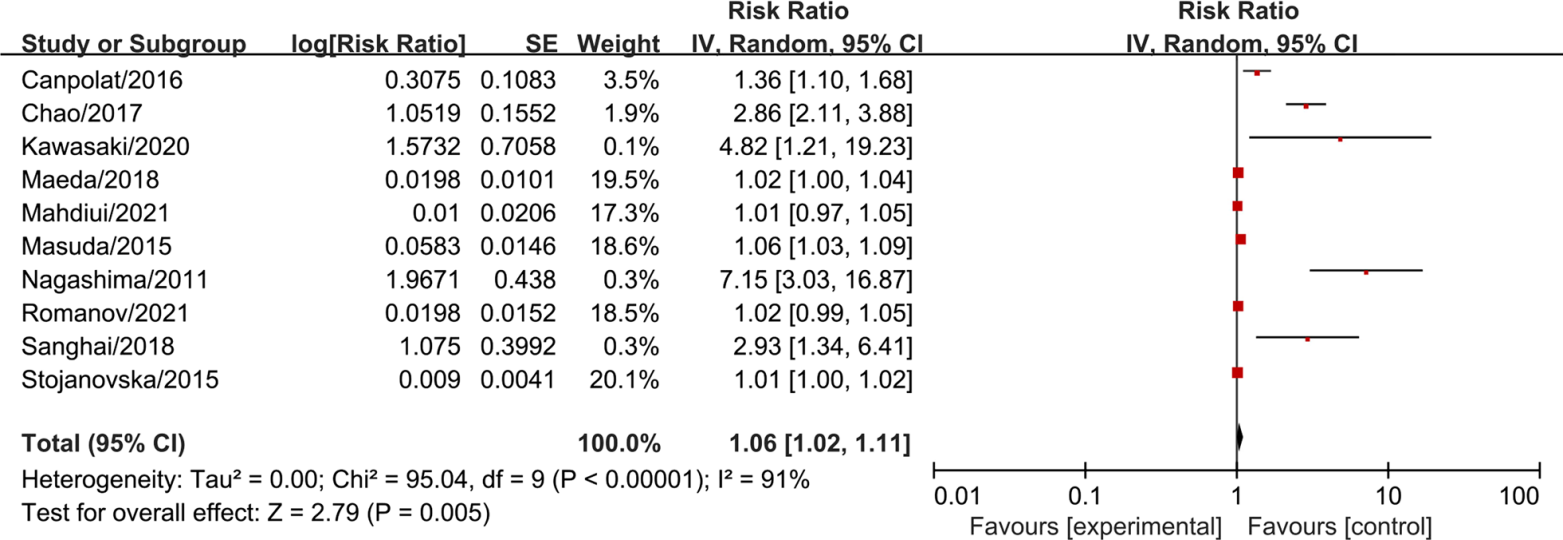
The forest plot of the association between the EAT amount and recurrence risk after catheter ablation in atrial fibrillation patients. The risk ratio (RR) is used to evaluate the association

**Fig. 3:**
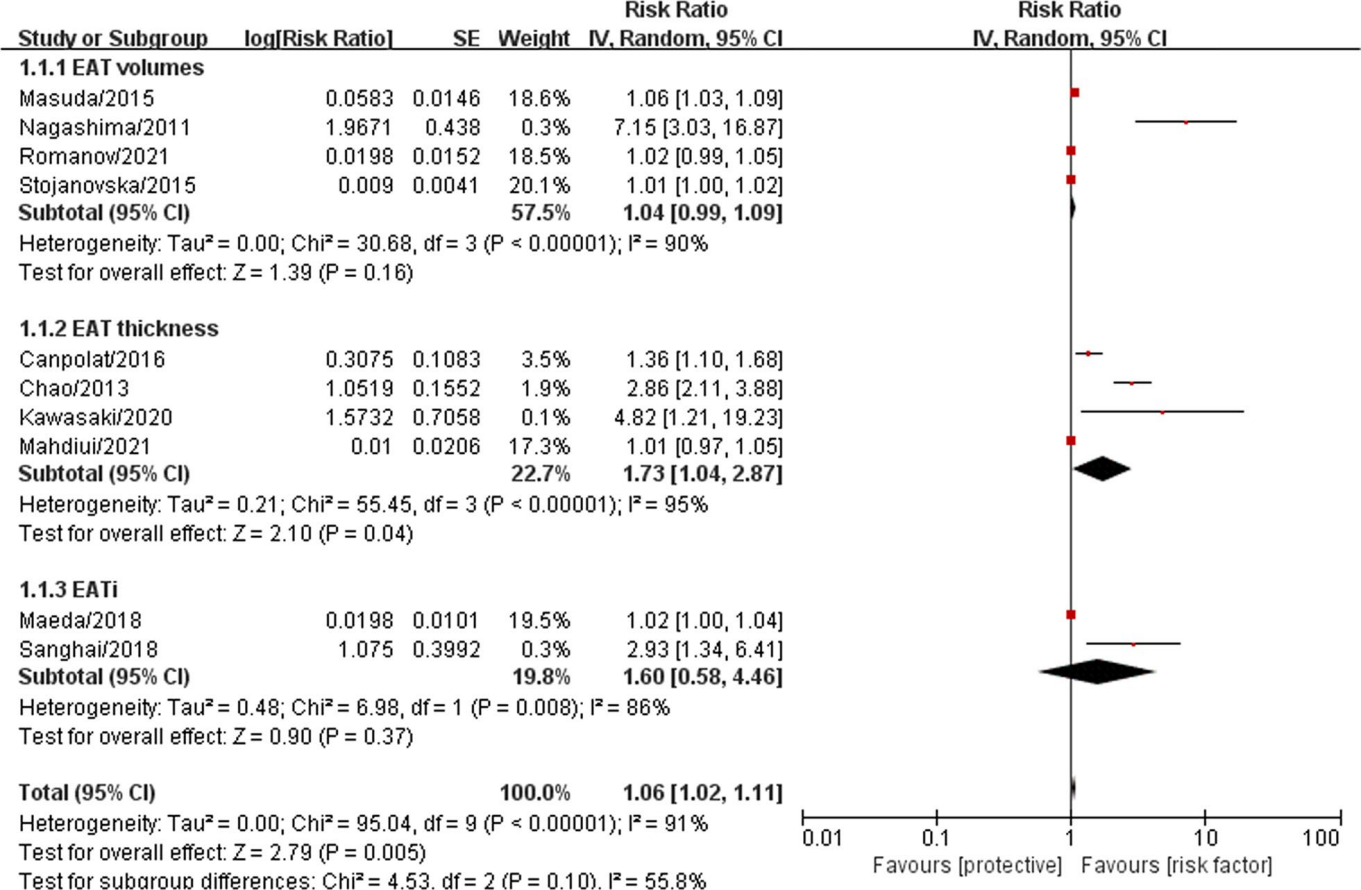
The forest plot of subgroup analysis (EAT related parameters) for the association between the EAT amount and recurrence risk after catheter ablation in atrial fibrillation patients. The risk ratio (RR) is used to evaluate the association

Totally, 1840 AF patients from the included studies were pooled together for the meta-analysis to investigate the association between EAT amount and AF recurrence after catheter ablation. The result showed that EAT was associated with higher recurrence risk in AF patients after catheter ablation with statistic difference (RR = 1.06, 95% CI 1.02–1.11, P = 0.005, forest plot shown in Fig. 2). Further subgroup analysis result suggested that EAT related thickness was the risk factor for AF recurrent after catheter ablation (RR = 1.73, 95% CI 1.04–2.87, P = 0.040, forest plot shown in Fig. 3), however, we did not confirm EAT related volume (RR = 1.04, 95% CI 0.99–1.09, P = 0.160, forest plot shown in Fig. 3) and EAT related index (RR = 1.60, 95% CI 0.58–4.46, P = 0.370, forest plot shown in Fig. 3) were significant associated with higher recurrence risk in AF patients after catheter ablation.

**Fig. 4:**
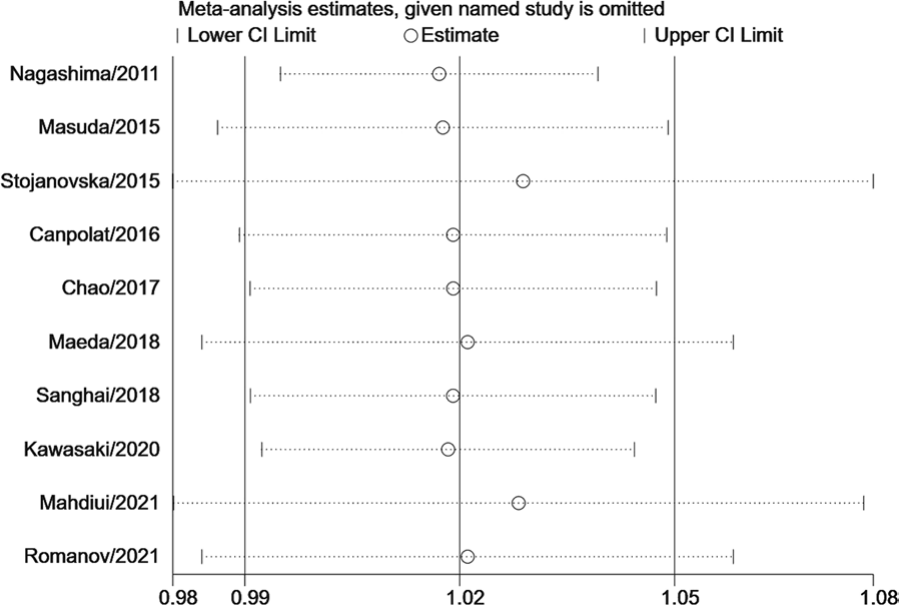
The sensitivity analysis for the association between the EAT amount and recurrence risk after catheter ablation in atrial fibrillation patients. Each branch represents the named study that was omitted; the merged effect size of the studies that remained

**Fig. 5:**
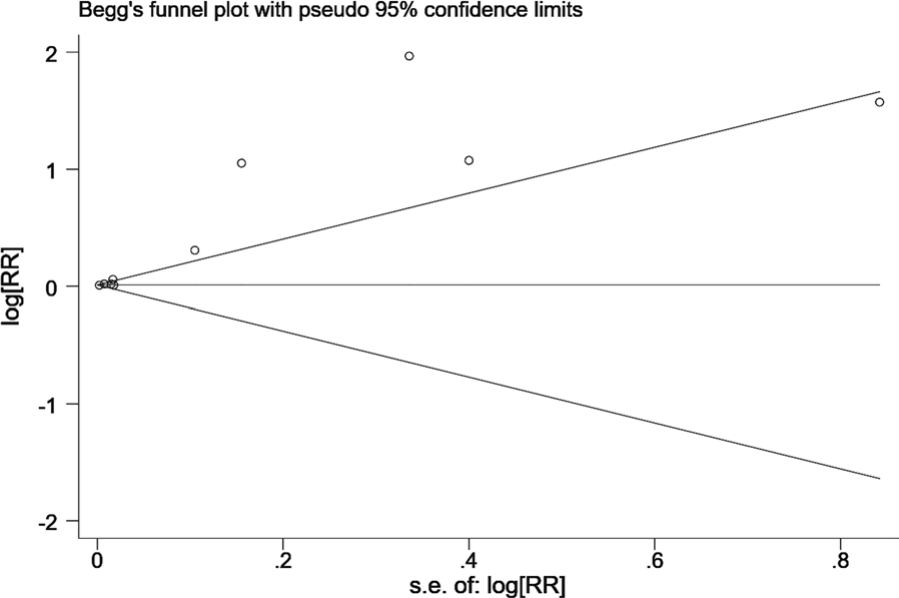
A funnel plot for the results of publish bias

For further investigating the effect of inter-study clinical heterogeneity on the final results, we performed four subgroup analysis according to the above methodological description. Subgroup meta-analysis demonstrated that EAT amount was associated with more significant higher-recurrence risk in the study with population average age under 60 years old (RR = 2.01, 95% CI 1.18–3.44, P = 0.010) compared with those population average age over 60 years old study (RR = 1.03, 95% CI 1.00–1.05, P = 0.070), as shown in Additional file 4: Fig. S1. These results suggested that EAT amount may play an important role in the AF recurrent after catheter ablation in younger AF patients. In the subgroup analysis of different regions, we found that the association between EAT and AF recurrence was significantly stronger in the Asian region group (RR = 1.25, 95% CI 1.10–1.43, P < 0.001) compared with Non-Asian region group (RR = 1.02, 95% CI 0.99–1.06, P = 0.210), as shown in Additional file 5: Fig. S2. In contrast to the studies with short-term follow-up (RR = 2.96, 95% CI 0.99–8.85, P = 0.050), studies with long-term follow-up (> 1 year) tend to observed a significant result (RR = 1.06, 95% CI 1.01–1.11, P = 0.020), as shown in Additional file 6: Fig. S3. Compared to the studies with small sample size (RR = 1.04, 95% CI 0.99–1.11, P = 0.150), large sample size studies were easier to obtain a result with significant differences (RR = 1.25, 95% CI 1.09–1.43, P = 0.001), as shown in Additional file 7: Fig. S4. We also made a sensitivity analysis to test the robustness of our final results and it’s statistically reliable, as shown in Fig. 4. Finally, we pooled the publication bias analysis by Egger’s test, as shown in Fig. 5. The results showed that there was a publication bias in the meta-analysis.

## Discussion

Our meta-analysis based on observational retrospective studies that pooled together 10 studies with 1840 AF patients. The result showed that EAT was associated with higher risk of AF recurrence after catheter ablation. Further analysis demonstrated that this association was more common in Asian AF population, younger AF patients (age < 60 years old), and long-term follow-up (> 1 year).

Previous meta-analysis have reported the association between EAT and AF recurrence after ablation based on comparing the relevant epicardial fat parameters (total EFT volume, LA-EFT volume, and EFT thickness) between the AF recurrence group and non-recurrence group. The results of previous meta-analysis showed that LA-EFT, EFT thickness, and total EFT volumes were increased in AF recurrent subjects [26]. Since their study did not adjust for other risk factors which would affect AF recurrence after catheter ablation, the association between EAT and AF recurrence risk was still not exact. However, our study merge the RR or HR of relevant epicardial fat parameters, which were fully adjusted by multivariable analysis in their study. Therefore, our research may provide more intuitive and convincing pieces of evidences for EAT contribute to AF recurrence after catheter ablation. Moreover, we confirmed that this association was more common in Asian population, younger AF patients. This will provide evidence for further stratification of AF patient who preparing for catheter ablation.

Obesity is a worldwide healthcare problem, which affected 670 million adults in 2016 around the world [27]. Globally, the prevalence of obesity in adults has almost tripled since 1975, and the prevalence of obesity by more than ten times in some middle-income countries [28]. Obesity proved to be an independent risk factor for AF [29]. Specifically, every 5-unit increment in body mass index was found to confer an additional 19% to 29% risk of AF incident, a 10% risk of post-operative AF, and a 13% risk of post-ablation AF [30]. However, the relationship between obesity and AF was not clear until the further exploration of EAT [31].

EAT was unique fat tissue that distinguishes from other depots of visceral fat. Epicardium shares an unobstructed microcirculation with the underlying myocardium without distinct boundaries, and produces cytokines that nourish the heart in healthy conditions [31]. However, in the states of systemic inflammation, the inflammation of EAT can act in a paracrine manner to influence the structure and function of neighboring myocardium tissues [31]. Currently, the mechanisms of EAT on AF are still under early exploration, potential mechanisms could be summarized as myocardial fatty infiltration, paracrine-associated pro-myocardial fibrosis, and inflammation. Mahajan R et al. reported that sustained obesity contribute to atrial remodeling characterized by left atrial enlargement, conduction abnormalities, fractionated electrograms, interstitial atrial fibrosis, and increased propensity for AF [32]. Further study detected that sustained obesity were associated with EAT infiltration of contiguous atrial myocardium [32, 33]. Such direct fatty infiltration separating myocytes could directly result in conduction slowing, promoting conduction heterogeneity that contribute to AF [34]. The paracrine of EAT is another important mechanism for AF formation. The increase in body weight was paralleled by an increase in aldosterone production, which was synthesized excessively both by adrenal gland and by adipocytes in obese people [35, 36]. Increased mineralogical corticosteroid signaling plays a key role in the transition of adipose tissue from a nourishing to pro-inflammatory state and was accompanied by the secretion of proinflammatory cytokines and by increased traffic of profibrotic mesenchymal stem cells from the epicardium [37, 38]. More important, Abe I et al. detected fibrotic remodeling and cytokines/chemokines (interleukin-6, monocyte chemoattractant protein-1, tumor necrosis factor-α, vascular endothelial growth factor, and matrix metalloproteinase-2 and matrix metalloproteinase-9) in peri-left atrial EAT were associated with atrial myocardial fibrosis [39].

EAT maybe a tool for identify those individuals more susceptible to recurrent after catheter ablation. However, the evaluation indexes of EAT in current studies are not uniform, EAT volume, EAT thickness, EAT volume index or LA EAT volume/thickness are common in present studies. Early studies (published before 2015 years) were focused on the volume or thickness of EAT, while subsequent studies (published after 2015 years) were turn to the index parameter related to EAT. In terms of the thickness of EAT, Chao et al. and Canpolat et al. indicated that 6.9 mm may be a good cut-off value for predicting the recurrence after ablation [13, 14]. Maeda et al. introduced the concept of EAT volume index, which is calculated as EAT volume/body surface area, this parameter can be effectively adjusted for individual size differences (BMI, or congenital difference) [25]. Another approach was measuring the relative amount of periatrial left atrium EAT, rather than the total EAT related parameters. Kawasaki et al. included three EAT related parameters (total EAT volume, periatrial EAT volume, periatrial to total EAT volume ratio) and found that periatrial to total EAT volume ratio ≥ 17.1% was of higher value for predicting AF recurrence after ablation than other indicators [16]. However, it seems that the effect of individual differences on EAT has not been considered based on this parameter (periatrial to total EAT volume ratio). Our study found that EAT related thickness (RR = 1.73, 95% CI 1.04–2.87, P = 0.040) was strongly associated with high recurrence risk after catheter ablation with statistical difference. EAT related thickness seems to be the marker most strongly associated with a greater risk of AF recurrences after catheter ablation, we hope more evidences will be provided in further prospective observational studies. In contrast to the EAT related thickness, EAT related volume (RR = 1.04, 95% CI 0.99–1.09, P = 0.160) and EAT related index (RR = 1.60, 95% CI 0.58–4.46, P = 0.370) have not showed a significant association with high recurrence risk. Due to the limited number of studies on EAT related index, there is still lack of full confirmation for the significant effect of EAT related index and EAT related volume on AF recurrence risk. Future researches need to effectively evaluate the existing EAT related parameters to determine the best indicators. Furthermore, making unified standard for EAT evaluation, and including it in risk stratification for clinical practice.

### Study limitation

Some limitations should be taken into account in our study. First, all included studies for meta-analysis were retrospective studies, which may exist some bias compared with prospective cohort studies. Our study confirmed that there was a certain publication bias in the present study. Future prospective cohort studies are needed to provide more convincing evidence on this topic. Second, the sample size was not large enough among each study, and a total of 1840 AF subjects were included in the present meta-analysis, however, it’s the largest study for investigating the association between EAT and AF recurrence after catheter ablation. Third, although we have made subgroup analysis across EAT related parameters, population average age, region, follow-up duration, and the study sample size, however, the result of our meta-analysis remained a high heterogeneity. We refer to previous two meta-analysis of this area, there were also a nonnegligible heterogeneity in their studies [12, 26]. We speculated that the greater clinical heterogeneity among each study may account for this result.

## Conclusion

Our study demonstrated that EAT was associated with a higher risk of AF recurrence after catheter ablation. EAT related thickness seems to be the marker most strongly associated with a greater risk of AF recurrences after catheter ablation. It is necessary to perform an evaluation of EAT for AF individuals before catheter ablation. In addition, we hope future researches will provide more evidence for making a unified standard for EAT evaluation, and include it in risk stratification for predicting AF recurrence after catheter ablation.

## Supplementary Information

**Additional file1**: PRISMA-P (Preferred Reporting Items for Systematic Review and Meta-Analysis Protocols) 2015 checklist: recommended items addressed in our systematic review and meta-analysis.

**Additional file2**: Search strategy.

**Additional file3**: Quality evaluation scale for prevalence studies.

**Additional file4: Fig. S1.** The forest plot of subgroup analysis (population average age) for the association between the EAT amount and recurrence risk after catheter ablation in atrial fibrillation patients. The risk ratio (RR) is used to evaluate the association.

**Additional file5: Fig. S2.** The forest plot of subgroup analysis (The regions) for the association between the EAT amount and recurrence risk after catheter ablation in atrial fibrillation patients. The risk ratio (RR) is used to evaluate the association. (PNG 16 KB)

**Additional file6: Fig. S3.** The forest plot of subgroup analysis (The follow-up duration) for the association between the EAT amount and recurrence risk after catheter ablation in atrial fibrillation patients. The risk ratio (RR) is used to evaluate the association.

**Additional file7: Fig. S4.** The forest plot of subgroup analysis (The study size) for the association between the EAT amount and recurrence risk after catheter ablation in atrial fibrillation patients. The risk ratio (RR) is used to evaluate the association.

## Data Availability

All data generated or analysed during this study are included in this published article [and its supplementary information files].

## Abbreviations

AF: Atrial fibrillation
CI: Confidence intervals
RR: Relative risk
HR: Hazard ratio
aHR: Adjusted hazard ratio
EAT: Epicardial adipose tissue
EFT: Epicardial fat tissue

## References

[1] Lippi G, Sanchis-Gomar F, Cervellin G (2021). Global epidemiology of atrial fibrillation: an increasing epidemic and public health challenge. Int J Stroke.

[2] Wendelboe AM, Raskob GE (2016). Global burden of thrombosis: epidemiologic aspects. Circ Res.

[3] Calkins H, Hindricks G, Cappato R (2018). 2017 HRS/EHRA/ECAS/APHRS/SOLAECE expert consensus statement on catheter and surgical ablation of atrial fibrillation. Europace.

[4] Poole JE, Bahnson TD, Monahan KH (2020). CABANA investigators and ECG rhythm core lab. Recurrence of atrial fibrillation after catheter ablation or antiarrhythmic drug therapy in the CABANA trial. J Am Coll Cardiol.

[5] Deng H, Bai Y, Shantsila A, Fauchier L, Potpara TS, Lip GYH (2017). Clinical scores for outcomes of rhythm control or arrhythmia progression in patients with atrial fibrillation: a systematic review. Clin Res Cardiol.

[6] Iacobellis G, Bianco AC (2011). Epicardial adipose tissue: emerging physiological, pathophysiological and clinical features. Trends Endocrinol Metab.

[7] Chaowalit N, Somers VK, Pellikka PA, Rihal CS, Lopez-Jimenez F (2006). Subepicardial adipose tissue and the presence and severity of coronary artery disease. Atherosclerosis.

[8] Al Chekakie MO, Welles CC, Metoyer R, Ibrahim A, Shapira AR, Cytron J (2010). Pericardial fat is independently associated with human atrial fibrillation. J Am Coll Cardiol.

[9] Shin SY, Yong HS, Lim HE, Na JO, Choi CU, Choi JI (2011). Total and interatrial epicardial adipose tissues are independently associated with left atrial remodeling in patients with atrial fibrillation. J Cardiovasc Electrophysiol.

[10] Wong CX, Abed HS, Molaee P, Nelson AJ, Brooks AG, Sharma G (2011). Pericardial fat is associated with atrial fibrillation severity and ablation outcome. J Am Coll Cardiol.

[11] Mahabadi AA, Lehmann N, K€alsch H, Bauer M, Dykun I, Kara K (2014). Association of epicardial adipose tissue and left atrial size on non-contrast CT with atrial fibrillation: the Heinz Nixdorf Recall Study. Eur Heart J Cardiovasc Imag.

[12] Gaeta M, Bandera F, Tassinari F, Capasso L, Cargnelutti M, Pelissero G, Malavazos AE, Ricci C (2017). Is epicardial fat depot associated with atrial fibrillation?. Syst Rev Meta-Anal Eur.

[13] Canpolat U, Aytemir K, Yorgun H, Asil S, Dural M, Özer N (2016). The impact of echocardiographic epicardial fat thickness on outcomes of cryoballoon-based atrial fibrillation ablation. Echocardiography.

[14] Chao TF, Hung CL, Tsao HM, Lin YJ, Yun CH, Lai YH, Chang SL, Lo LW, Hu YF, Tuan TC, Chang HY, Kuo JY, Yeh HI, Wu TJ, Hsieh MH, Yu WC, Chen SA (2013). Epicardial adipose tissue thickness and ablation outcome of atrial fibrillation. PLoS ONE.

[15] Sanghai SR, Sardana M, Hansra B, Lessard DM, Dahlberg ST, Aurigemma GP, Fitzgibbons TP, McManus DD (2018). Indexed left atrial adipose tissue area is associated with severity of atrial fibrillation and atrial fibrillation recurrence among patients undergoing catheter ablation. Front Cardiovasc Med.

[16] Kawasaki M, Yamada T, Furukawa Y, Morita T, Tamaki S, Kida H, Sakata Y, Fukunami M (2020). Are cardiac sympathetic nerve activity and epicardial adipose tissue associated with atrial fibrillation recurrence after catheter ablation in patients without heart failure?. Int J Cardiol.

[17] El Mahdiui M, Simon J, Smit JM, Kuneman JH, van Rosendael AR, Steyerberg EW, van der Geest RJ, Száraz L, Herczeg S, Szegedi N, Gellér L, Delgado V, Merkely B, Bax JJ, Maurovich-Horvat P (2021). Posterior left atrial adipose tissue attenuation assessed by computed tomography and recurrence of atrial fibrillation after catheter ablation. Circ Arrhythm Electrophysiol.

[18] Romanov A, Minin S, Nikitin N, Ponomarev D, Shabanov V, Losik D, Steinberg JS (2021). The relationship between global cardiac and regional left atrial sympathetic innervation and epicardial fat in patients with atrial fibrillation. Ann Nucl Med.

[19] Tu Y-K, Greenwood DC (2012). Modern methods for epidemiology.

[20] Moher D, Liberati A, Tetzlaff J, Altman DG (2009). PRISMA group. Preferred reporting items for systematic reviews and meta-analyses: the PRISMA statement. PLoS Med.

[21] Stang A (2010). Critical evaluation of the Newcastle-Ottawa scale for the assessment of the quality of nonrandomized studies in meta-analyses. Eur J Epidemiol.

[22] Nagashima K, Okumura Y, Watanabe I, Nakai T, Ohkubo K, Kofune T, Kofune M, Mano H, Sonoda K, Hirayama A (2011). Association between epicardial adipose tissue volumes on 3-dimensional reconstructed CT images and recurrence of atrial fibrillation after catheter ablation. Circ J.

[23] Masuda M, Mizuno H, Enchi Y, Minamiguchi H, Konishi S, Ohtani T, Yamaguchi O, Okuyama Y, Nanto S, Sakata Y (2015). Abundant epicardial adipose tissue surrounding the left atrium predicts early rather than late recurrence of atrial fibrillation after catheter ablation. J Interv Card Electrophysiol.

[24] Stojanovska J, Kazerooni EA, Sinno M, Gross BH, Watcharotone K, Patel S, Jacobson JA, Oral H (2015). Increased epicardial fat is independently associated with the presence and chronicity of atrial fibrillation and radiofrequency ablation outcome. Eur Radiol.

[25] Maeda M, Oba K, Yamaguchi S, Arasaki O, Sata M, Masuzaki H, Shimabukuro M (2018). Usefulness of epicardial adipose tissue volume to predict recurrent atrial fibrillation after radiofrequency catheter ablation. Am J Cardiol.

[26] Sepehri Shamloo A, Dagres N, Dinov B, Sommer P, Husser-Bollmann D, Bollmann A, Hindricks G, Arya A (2019). Is epicardial fat tissue associated with atrial fibrillation recurrence after ablation? A systematic review and meta-analysis. Int J Cardiol Heart Vasc.

[27] NCD Risk Factor Collaboration (NCD-RisC) (2017). Worldwide trends in body-mass index, underweight, overweight, and obesity from 1975 to 2016: a pooled analysis of 2416 population-based measurement studies in 128·9 million children, adolescents, and adults. Lancet.

[28] Wang L, Zhou B, Zhao Z, Yang L, Zhang M, Jiang Y, Li Y, Zhou M, Wang L, Huang Z, Zhang X, Zhao L, Yu D, Li C, Ezzati M, Chen Z, Wu J, Ding G, Li X (2021). Body-mass index and obesity in urban and rural China: findings from consecutive nationally representative surveys during 2004–18. Lancet.

[29] Lavie CJ, Pandey A, Lau DH, Alpert MA, Sanders P (2017). Obesity and atrial fibrillation prevalence, pathogenesis, and prognosis: effects of weight loss and exercise. J Am Coll Cardiol.

[30] Wong CX, Sullivan T, Sun MT, Mahajan R, Pathak RK, Middeldorp M, Twomey D, Ganesan AN, Rangnekar G, Roberts-Thomson KC, Lau DH, Sanders P (2015). Obesity and the risk of incident, post-operative, and post-ablation atrial fibrillation: a meta-analysis of 626,603 individuals in 51 studies. JACC Clin Electrophysiol.

[31] Packer M (2018). Epicardial adipose tissue may mediate deleterious effects of obesity and inflammation on the myocardium. J Am Coll Cardiol.

[32] Mahajan R, Lau DH, Brooks AG, Shipp NJ, Manavis J, Wood J, Finnie J, Samuel C, Royce S, Twomey D, Thanigaimani S, Kalman JM, Sanders P (2015). Electrophysiological, electroanatomical and structural remodeling of the atria as a consequence of sustained obesity. J Am Coll Cardiol.

[33] Nalliah CJ, Bell JR, Raaijmakers AJA, Waddell HM, Wells SP, Bernasochi GB, Montgomery MK, Binny S, Watts T, Joshi SB, Lui E, Sim CB, Larobina M, O'Keefe M, Goldblatt J, Royse A, Lee G, Porrello ER, Watt MJ, Kistler PM, Sanders P, Delbridge LMD, Kalman JM (2020). Epicardial adipose tissue accumulation confers atrial conduction abnormality. J Am Coll Cardiol.

[34] Mahajan R, Kuklik P, Grover S, Brooks AG, Wong CX, Sanders P, Selvanayagam JB (2013). Cardiovascular magnetic resonance of total and atrial pericardial adipose tissue: a validation study and development of a 3 dimensional pericardial adipose tissue model. J Cardiovasc Magn Reson.

[35] Bentley-Lewis R, Adler GK, Perlstein T, Seely EW, Hopkins PN, Williams GH, Garg R (2007). Body mass index predicts aldosterone production in normotensive adults on a high-salt diet. J Clin Endocrinol Metab.

[36] Huby AC, Antonova G, Groenendyk J, Gomez-Sanchez CE, Bollag WB, Filosa JA, Belin de Chantemèle EJ (2015). Adipocyte-derived hormone leptin is a direct regulator of aldosterone secretion, which promotes endothelial dysfunction and cardiac fibrosis. Circulation.

[37] Qian C, Schoemaker RG, van Gilst WH, Roks AJ (2009). The role of the renin-angiotensin-aldosterone system in cardiovascular progenitor cell function. Clin Sci (Lond).

[38] Even SE, Dulak-Lis MG, Touyz RM, Nguyen Dinh Cat A (2014). Crosstalk between adipose tissue and blood vessels in cardiometabolic syndrome: implication of steroid hormone receptors (MR/GR). Horm Mol Biol Clin Investig.

[39] Abe I, Teshima Y, Kondo H (2018). Association of fibrotic remodeling and cytokines/chemokines content in epicardial adipose tissue with atrial myocardial fibrosis in patients with atrial fibrillation. Heart Rhythm.

