# Supplementary material for: Epicardial adipose tissue is associated with higher recurrence risk after catheter ablation in atrial fibrillation patients: a systematic review and meta-analysis
Source: BMC Cardiovasc Disord. 2022 Jun 11;22:264. doi: 10.1186/s12872-022-02703-9 (PMC9188706; doi:10.1186/s12872-022-02703-9)
Supplement: Supplementary file 2 — Additional file2: Search strategy. [file 12872_2022_2703_MOESM2_ESM.docx]

***Search strategy：***

**Pubmed:**

**#1:(epicardial adipose tissue[Title/Abstract]) OR (epicardial fat[Title/Abstract]) OR (EAT[Title/Abstract]) OR (EFT[Title/Abstract])**

**#2:(****atrial fibrillation[Title/Abstract])OR(AF[Title/Abstract])**

**#3:(catheterablation[Title/Abstract])OR(radiofrequencyablation[Title/Abstract])**

**#4: #1 AND #2 AND #3**

**Embase:**

**#1: 'epicardial adipose tissue'/exp OR 'epicardial fat':ti,ab,kw OR 'EAT':ti,ab,kw OR 'EAT':ti,ab,kw**

**#2: atrial fibrillation:ti,ab,kw OR AF:ti,ab,kw**

**#3: 'catheter ablation':ti,ab,kw OR 'radiofrequencyablation':ti,ab,kw**

**#4: #1 AND #2 AND #3**

**Cochrane Library:**

**#1: MeSH descriptor:[epicardial adipose tissue] explode all trees**

**#2: (atrial fibrillation):ti,ab,kw OR (AF):ti,ab,kw**

**#3:(catheter ablation):ti,ab,kw OR (radiofrequencyablation):ti,ab,kw**

**#4: #1 AND #2 AND #3**
