# Supplementary material for: Epicardial adipose tissue is associated with higher recurrence risk after catheter ablation in atrial fibrillation patients: a systematic review and meta-analysis
Source: BMC Cardiovasc Disord. 2022 Jun 11;22:264. doi: 10.1186/s12872-022-02703-9 (PMC9188706; doi:10.1186/s12872-022-02703-9)
Supplement: Supplementary file 3 — Additional file3: Quality evaluation scale for prevalence studies. [file 12872_2022_2703_MOESM3_ESM.docx]

|  | **Selection** | | | | **Comparability** | **Outcome** | | | **[Quality](javascript:;) evaluation** |
| --- | --- | --- | --- | --- | --- | --- | --- | --- | --- |
|  | **Exposed cohort** | **Non exposed cohort** | **Ascertainment of exposure** | **Outcome of interest** | **Cohorts comparability** | **Outcome aassessment** | **Follow-up for outcomes to occur** | **Adequacy of follow up** |  |
| **Canpolat/2016** | **Representativeness** | **Representativeness** | **secure record** | **yes** | **yes** | **yes** | **yes** | **yes** | **high** |
| **Chao/2017** | **Representativeness** | **Representativeness** | **secure record** | **yes** | **yes** | **yes** | **yes** | **yes** | **high** |
| **Sanghai/2018** | **Representativeness** | **Representativeness** | **secure record** | **yes** | **yes** | **yes** | **yes** | **yes** | **high** |
| **Kawasaki/2020** | **Representativeness** | **Representativeness** | **secure record** | **yes** | **yes** | **yes** | **yes** | **yes** | **high** |
| **Mahdiui/2021** | **Representativeness** | **Representativeness** | **secure record** | **yes** | **yes** | **yes** | **yes** | **yes** | **high** |
| **Romanov/2021** | **Representativeness** | **Representativeness** | **secure record** | **yes** | **yes** | **yes** | **yes** | **No statement** | **[moderate](file:///C:/Program%2520Files%2520(x86)/Youdao/Dict/8.7.0.0/resultui/html/index.html#/javascript:;)** |
| **Maeda/2018** | **Representativeness** | **Representativeness** | **secure record** | **yes** | **yes** | **yes** | **yes** | **yes** | **high** |
| **Nagashima/2011** | **Representativeness** | **Representativeness** | **secure record** | **yes** | **yes** | **yes** | **yes** | **yes** | **high** |
| **Masuda/2015** | **Representativeness** | **Representativeness** | **secure record** | **yes** | **yes** | **yes** | **yes** | **yes** | **high** |
| **Stojanovska/2015** | **Representativeness** | **Representativeness** | **secure record** | **yes** | **yes** | **yes** | **yes** | **yes** | **high** |

***Supplementary Table 3 NOS for each study***
