# Supplementary figures and images for: Epicardial adipose tissue is associated with higher recurrence risk after catheter ablation in atrial fibrillation patients: a systematic review and meta-analysis
Source: BMC Cardiovasc Disord. 2022 Jun 11;22:264. doi: 10.1186/s12872-022-02703-9 (PMC9188706; doi:10.1186/s12872-022-02703-9)

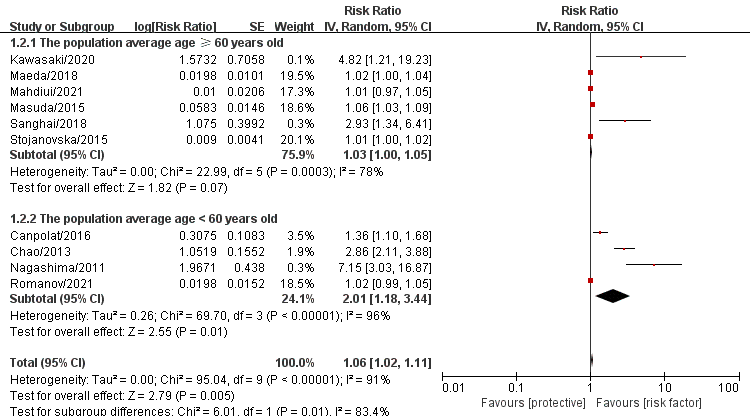

Supplement: Supplementary file 4 — Additional file4: Fig. S1. The forest plot of subgroup analysis (population average age) for the association between the EAT amount and recurrence risk after catheter ablation in atrial fibrillation patients. The risk ratio (RR) is used to evaluate the association. [file 12872_2022_2703_MOESM4_ESM.png]

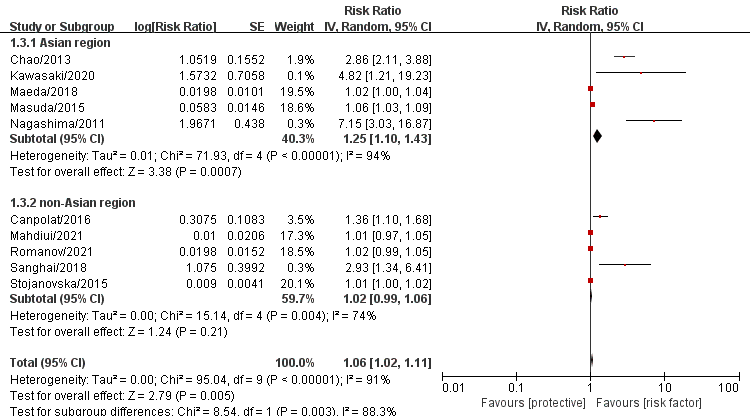

Supplement: Supplementary file 5 — Additional file5: Fig. S2. The forest plot of subgroup analysis (The regions) for the association between the EAT amount and recurrence risk after catheter ablation in atrial fibrillation patients. The risk ratio (RR) is used to evaluate the association. (PNG 16 KB) [file 12872_2022_2703_MOESM5_ESM.png]

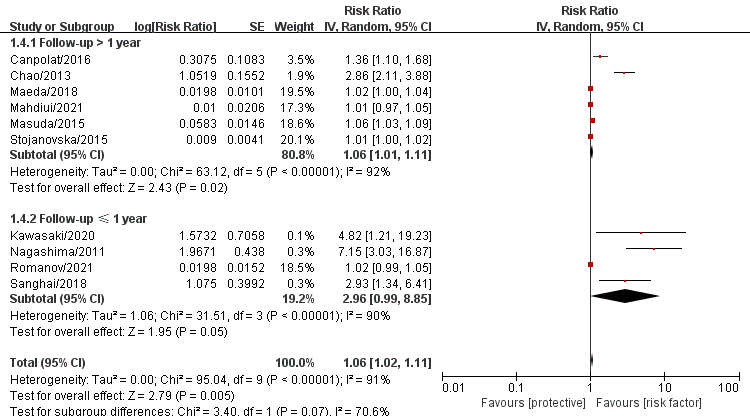

Supplement: Supplementary file 6 — Additional file6: Fig. S3. The forest plot of subgroup analysis (The follow-up duration) for the association between the EAT amount and recurrence risk after catheter ablation in atrial fibrillation patients. The risk ratio (RR) is used to evaluate the association. [file 12872_2022_2703_MOESM6_ESM.png]

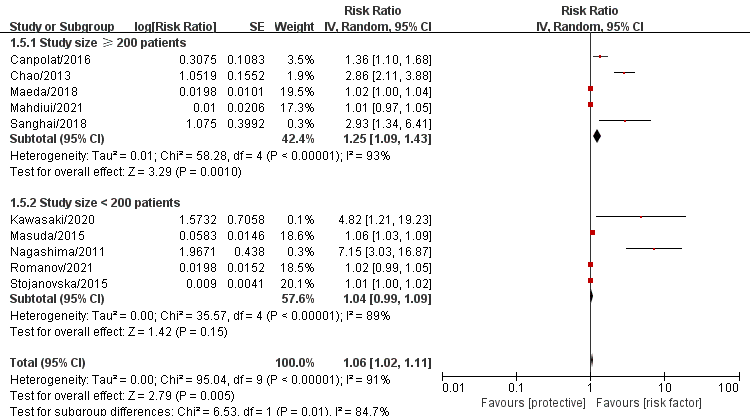

Supplement: Supplementary file 7 — Additional file7: Fig. S4. The forest plot of subgroup analysis (The study size) for the association between the EAT amount and recurrence risk after catheter ablation in atrial fibrillation patients. The risk ratio (RR) is used to evaluate the association. [file 12872_2022_2703_MOESM7_ESM.png]
